# Road Salt versus Urban Snow Effects on Lake Microbial Communities

**DOI:** 10.3390/microorganisms10040803

**Published:** 2022-04-12

**Authors:** Isabelle B. Fournier, Connie Lovejoy, Warwick F. Vincent

**Affiliations:** 1 Departement de Biologie, Institut de Biologie Integrative et des Systemes (IBIS), Centre D’etudes Nordiques (CEN), Universite Laval, Quebec City, QC G1V 0A6, Canada; connie.lovejoy@bio.ulaval.ca (C.L.); warwick.vincent@bio.ulaval.ca (W.F.V.); 2 CentrEau, Universite Laval, Quebec City, QC G1V 0A6, Canada; 3 Quebec Ocean, Universite Laval, Quebec City, QC G1V 0A6, Canada

**Keywords:** bacteria, chloride, microbial eukaryotes, plankton, urban lakes, road salts, microcosms

## Abstract

Freshwater salinization is an ongoing concern for north temperate lakes; however, little is known about its impacts on microbial communities, particularly for bacteria. We tested the hypotheses that road de-icing salt induces changes in the microbial community structure of lake plankton, and that changes due to chloride would differ from those due to urban snowmelt because of additional chemicals in the snowmelt. In a laboratory incubator experiment, an overwintering plankton community in lake water was exposed for two weeks to either NaCl or municipal road snow with the same level of chloride. Microbial community structure as determined by 16S (prokaryotes) and 18S (eukaryotes) rRNA transcript analysis showed changes in response to the chloride-only enrichment, with some rare taxa becoming more prominent. Consistent with our hypothesis, the salt and the snow treatments induced different community changes. These results indicate that ecotoxicology assays based on a single salt addition may not reflect the in situ effects of salt-contaminated urban snow, and that the combined chemical effects of urban snowmelt require direct testing.

## 1. Introduction

Chloride concentrations are increasing in many northern freshwater lakes due to winter road salt usage. To date, few of the salt-affected lakes contain chloride concentrations beyond the guideline limit of 230 mg L^−1^ (USA and Quebec) or 120 mg L^−1^ (Canada) for chronic exposure of freshwater aquatic life, and most lake values are well below this limit [1]. Research on the effects of sodium chloride (NaCl, the most commonly used road salt) at environmentally relevant concentrations has mainly focused on zooplankton, amphibians and fish, while little attention has been accorded to microbial communities [2] despite their fundamental role in underpinning aquatic ecosystem function. Recent studies suggest that the phytoplankton responses to increased salinity mostly reflect the responses via zooplankton and top-down control, but also that there are taxon-specific responses at concentrations below the guidelines, which need to be further explored [3,4].

Sodium chloride can reach lakes via roadside snowmelt, which contains inorganic nutrients, metals and organic contaminants such as polycyclic aromatic hydrocarbons [5,6,7]. In many temperate lakes and rivers, the snowmelt period at the beginning of spring corresponds to peak chloride loads and concentrations [7,8]. Information is therefore needed to understand the sensitivity of the overwintering communities to this seasonal rise in NaCl, and associated road-derived contaminants. 

Our aim in the present study was to evaluate the response of planktonic microbial prokaryote and eukaryote communities to chloride at environmentally relevant concentrations, either as pure sodium chloride, or as a component of urban snowmelt that also contains many other chemicals. Specifically, our objective was to test the hypotheses that chloride induces a shift in the taxonomic structure of overwintering microbial communities in lakes, and that these changes are different to those induced by urban snowmelt due to the presence of other chemical stressors and microorganisms. To address these hypotheses, we sampled the overwintering plankton of a freshwater reservoir (Lake Saint-Charles, Quebec City, QC, Canada) and exposed subsamples to different chemical treatments in a 2 week controlled laboratory experiment. Microbial community structure (bacteria and microbial eukaryotes) was assessed at the beginning and at the end of the experiment by 16S and 18S rRNA transcript analysis.

## 2. Materials and Methods

**Figure 1:**
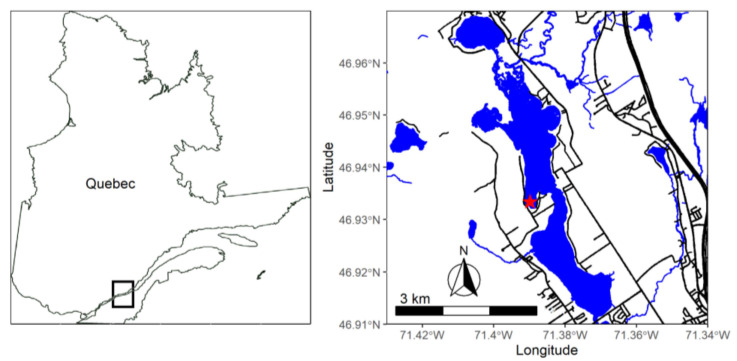
Localization of Lake Saint-Charles and of the sampling site (red star) of the overwintering microbial plankton.

Lake Saint-Charles (lat. 46.94° N, long. 71.39° W), the drinking water reservoir for Quebec City, Canada, was sampled 11 March 2016. The conductivity of this lake increases during spring (April–May) each year because of inputs from melting roadside snow that contains de-icing salts [7]. Surface water (0–50 cm) was sampled beneath the ice in a bay of the lake (Figure 1) for the experiment, and for chemical and microbial analyses. Physicochemical parameters (pH, conductivity, dissolved oxygen and temperature) at the time of sampling were measured in situ using a Hydrolab DS5X profiler (OTTHydroMet, Loveland, CO, USA). Road-influenced snow was obtained from the “de la Colline” snow depot (lat. 46.87° N, long. 71.35° W; 17 March 2016). This depot receives snow collected from roads, sidewalks, parking lots and other municipal spaces in the southern part of the Lake Saint-Charles watershed. This snow contains urban road residues, including salt. The snow was sampled at three locations in acid-washed (0.1 M HCl) storage boxes (Rubbermaid, Atlanta, GA, USA). The snow was left to melt in the dark at 4 °C and was used for the chemical analysis and the experiment. For the melted snow used in the experiment, the triplicates were pooled in equal volumes. Water and snow samples were analyzed for total nitrogen (TN), total phosphorus (TP), dissolved ions, dissolved organic carbon (DOC), alkalinity, and polycyclic aromatic hydrocarbons (PAHs). 

Total nitrogen (TN) and total phosphorus (TP) samples were acidified (H_2_SO_4_, 0.1% final concentration), then kept at 4 °C until analysis by colorimetric methods with, respectively, sulfanilamide and ascorbic acid reduction after persulfate digestion. Ion and dissolved organic carbon (DOC) samples were filtered through Milli-Q water pre-rinsed cellulose acetate filters (0.2 µm pore size, Advantec Micro Filtration Systems), then acidified (for cations only, HNO_3_ Trace Metal Grade, 0.2% final concentration) and kept at 4 °C until analysis. Anion concentrations were measured by ion chromatography (ICS-2000, Dionex, Sunnyvale, CA, USA), major cations by atomic emission spectroscopy (ICP-AES, Varian Vista AX), trace cations by mass spectroscopy (ICP-MS, Thermo X Series) and DOC by combustion catalytic oxidation (Shimadzu, Kyoto, Japan, TOC-5000A carbon analyzer calibrated with potassium biphthalate). Alkalinity (calcium carbonate equivalent) was determined with a titration by 0.02 N sulfuric acid [9]. Polycyclic aromatic hydrocarbons concentrations were determined by Maxxam Analytique Inc., Quebec, QC, Canada, using the QUE SOP-00207 protocol.

The lake water for the experiment was filtered through a 250 µm mesh to remove zooplankton, and then 2 L quantities were dispensed into each of the twelve 4 L LDPE Cubitainers^TM^ (Thermo Fisher Scientific). The Cubitainers were placed in a Sanyo environmental incubator at the Laboratoire aquatique de recherche en sciences environnementales et médicales (LARSEM) at Laval University (Quebec City, QC, Canada) under initial conditions of 4 °C, a photoperiod of 12:12, and a daytime irradiance cycle of 40–60–40 µmol photons m^−2^ s^−1^. This light and temperature regime was chosen to mimic lake conditions under the ice. Prior to initiating the experimental treatments, the incubator temperature was gradually increased to 15 °C, while irradiance was gradually increased to 80–150–80 µmol photons m^−2^ s^−1^ (same photoperiod as before) to simulate the onset of spring conditions. During the acclimation and the experiment, Cubitainers were rotated once a day in the incubator to ensure equal light exposure over time as well as manually inverted to resuspend plankton and renew oxygen. They were left open during the experiment to allow gas exchange.

For the experiment, the water was incubated at two Cl concentrations: the original lake water concentration of 20 mg Cl L^−1^, and ca. 50 mg Cl L^−1^, equal to a 2-fold increase in conductivity. This corresponded to the increase observed in the reservoir in spring [7]. Chloride was supplied either as NaCl (reagent grade) dissolved in Milli-Q water (hereafter referred to as the “Salt treatment”) or as melted urban snow containing Na^+^ and Cl^−^ as major ions (hereafter referred to as the “Snow treatment”). The snow was not sterilized by heating or filtration to avoid changing its chemical properties and potential toxicity [10], and also to allow the presence of snow microbiota that likely reach the lake in snowmelt runoff [11]. Phosphorus was added to the Salt and Snow treatments (+4 µg P L^−1^ final concentration, as KH_3_PO_4_) to prevent phosphorus limitation over the experiment, and was added on its own as a test for any effects of this nutrient addition (“P-Only treatment”). The experiment was conducted with triplicates: three Cubitainers were selected randomly for each treatment (*n* = 9), and three others were incubated with only Milli-Q addition (“Control”). Additions in all treatments represented 5% of the total volume. Two weeks after the start of the incubation, a subsample (300 mL) was taken from each Cubitainer for RNA amplicon analysis.

Each sample for amplicon analysis (ca. 1 L) was filtered through a 0.2 µm Sterivex^TM^ unit (Millipore, Burlington, MA, USA) that was then filled with RNAlater (Life Technologies, Carlsbad, CA, USA) and frozen at −80 °C until analysis. Nucleic acids were extracted from the Sterivex units using an AllPrep DNA/RNA Mini Kit (Qiagen, Hilden, Germany) and the Qiagen protocol modified as follows: (1) RLT + β-ME was added into the Sterivex units, which were incubated at 37 °C for 45 min. Lysozyme and proteinase k were then added into the units and they were incubated at 65 °C for 15 min. These steps were performed replacing Qiagen steps 1 and 2; (2) in the RNA purification phase, a DNA digestion step was added between Qiagen steps 8 and 9, with 10 µL of DNAse (Qiagen RNase free DNase set) diluted in 55 µL RDD Buffer added in the RNeasy spin columns and left for 15 min at room temperature. The RNeasy spin columns were then rinsed with Buffer RW1 as in step 8. Following testing for contaminating DNA, the extracted RNA was converted to cDNA with a High-Capacity cDNA Reverse Transcription Kit (Applied Biosystems-Ambion, Waltham, MA, USA). The transcription was carried out in 20 µL (10 µL RNA template, 2 µL RT Buffer 5X, 0.8 µL dntps, 2 µL RT primers, 1 µL transcriptase and 4.2 µL water) with the following thermal cycle: 25 °C for 10 min, 37 °C for 120 min and 85 °C for 5 min. The cDNA was stored at −80 °C until further analysis. The cDNA was amplified using two sets of primers modified with Illumina adaptors: (1) 515F/806R, which targets the V4 region of the 16S rRNA (prokaryotes, [12]); and (2) 572F/1009R, which targets the V4 region of the 18S rRNA (eukaryotes, [13]). PCR was carried out in a total volume of 25 µL (1 µL cDNA template, 5 µL PCR buffer (New England Biolabs), 1.25 µL reverse and forward primers, 0.5 µL mix dNTP, 0.25 µL Q5 High-Fidelity DNA Polymerase (New England Biolabs) and 15.75 µL water). Conditions of the PCR thermal cycling for each set of primers are presented in Table 1. PCR products were purified with ethanol and magnetic beads (Agencourt AMPure XP) and a second PCR was run to introduce the sample tags. This reaction had an initial denaturation temperature of 98 °C for 30 s, then 13 cycles of 10 s denaturation at 98 °C, 30 s annealing at 55 °C and 30 s elongation at 72 °C followed by a final extension of 4.5 min at 72 °C. The second amplicons were purified with beads as previously described, quantified with the Nanodrop 1000 (Thermo Fisher Scientific, Waltham, MA, USA), pooled in equimolar ratio and sequenced on an Illumina MiSeq at the IBIS/Laval University Plate-forme d’analyses génomiques (Quebec City, QC, Canada). We focused on ribosomal small subunit RNA transcripts rather than rRNA genes to provide a measure that is more representative of the active microbial community, and because this approach allowed a fine discrimination of community dynamics in this lake and others [14]. 

Forward and reverse read pairs were merged using bbmerge v37.36 [15], and the obtained merged reads were filtered with maximum expected errors of 1 [16]. Unique reads were identified as well as abundance and size filtered to discard chimera (300 bp) and singletons using vsearch [17]. USEARCH was then used to clustered reads at 98% similarity level (Operational Taxonomic Units, OTUs) for 18S and at 87% similarity level for 16S. We used mothur v1.39 [18] to assign the taxonomy of the most abundant sequence of each OTU based on the Protist Ribosomal Reference database (PR2, [19]) for 18S and SILVA 132 [20] for 16S. OTU tables were constructed with the number of reads per OTU in each sample.

Sequences identified as Charophyceae [21], rotifer, chloroplasts, and mitochondria were removed from further analysis. When relevant, unidentified OTUs were submitted to a BLAST search to the nr database of NCBI GenBank and identified to the closest match. The nucleotide sequence data reported are available in the NCBI database under accession number PRJNA681563.

The effects of the experimental factors (phosphorus enrichment and increase sodium chloride in either water or waste snow) on microbial plankton community structure were tested by a permutational multivariate analysis of variance (PERMANOVA, [22]), with a dummy variable representing the treatments, using the adonis function of the vegan package (v2.4.6, [23]). Principal coordinate analysis (PCoA) was conducted to visualize possible clustering of the samples with the pcoa function of the ape package (v5.1, [24]). This PCoA was based on a Bray–Curtis dissimilarity matrix using the vegdist function of the vegan package. The effects of experimental factors on individual taxonomic groups were tested with an analysis of variance using the aov function of the stats package (v3.5.1) on the square root of the relative abundance. When significant, differences were further investigate using Tukey’s Honest Significant Difference test with the TukeyHSD function of the stats package. All analyses were performed using the R software (v3.4.3, [25]).

## 3. Results

### 3.1. Properties of the Lake Water and Urban Snow

At the time of sampling, the 3 m water column at the sample site was covered by 50 cm of ice and 10 cm of snow. The air temperature was 0.5 °C, and the surface water (0–50 cm) had a temperature of 0.17 °C and conductivity of 125 µS cm^−1^, with 76% dissolved oxygen saturation and pH 7.18. The concentration of chloride was 20 mg L^−1^ (Table 2). 

Most of the measured chemical constituents in the snow were at higher concentrations than in the lake, with the exception of total nitrogen and potassium that were approximately half of lake water concentrations, and chloride values that were similar (Table 2). Three polycyclic aromatic hydrocarbons (PHAs), benzo(a)pyrene, fluoranthene and pyrene, were measured in the snow in concentrations higher than the CCME guidelines, which are, respectively, 0.015, 0.04, and 0.025 µg L^−1^, but were below detection in the lake water.

### 3.2. Microbial Plankton Responses

The 77,137 reads obtained for the lake sample of March 2016 using the 16S primer were classified into 307 OTUs. The dominant phyla were *Proteobacteria* (65% of total reads), comprising *Betaproteobacteria* (48% of total reads), *Gammaproteobacteria* (10%) and *Alphaproteobacteria* (7%); and *Bacteroidetes* (29%). The 18S primer yielded 44,163 reads, and 155 OTUs, and the dominant phyla were ochrophytes (45.2% of total reads), dominated by chrysophytes (45.1%), ciliates (32%), dinoflagellates (10%) and cercozoans (5%). By the end of the experiment, the Control had shifted from the original lake community. 

**Figure 2:**
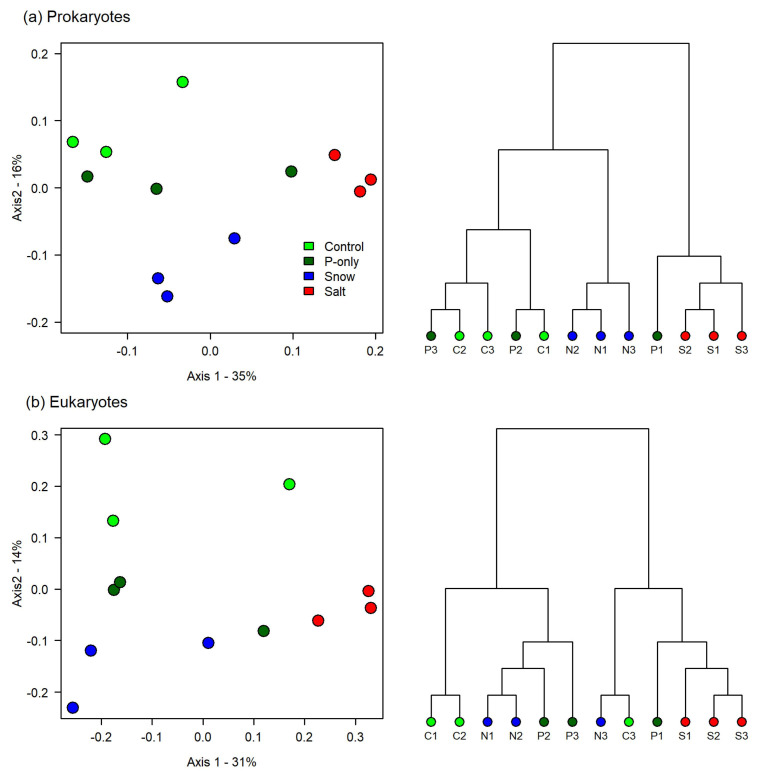
Principal component analysis (PCoA) for: (**a**) prokaryotes; (**b**) eukaryotes. Each point is a replicate. C = Control; P = P-Only; N = Snow; S = Salt.

The experimental treatments resulted in changes in both the prokaryotic and eukaryotic communities and explained 25% and 77% of the variation for these two groups, respectively (PERMANOVA; *F* = 2.50 and *F* = 2.14; *p* = 0.002 and *p* = 0.007). A PCoA constructed from a Bray–Curtis dissimilarity matrix revealed separate clusters for each treatment, with the Salt treatment separating from the others along the first axis (Figure 2). The first axis explained 35% and 31% of the variation, while the second axis explained 16% and 14% of the variation for the prokaryotes and eukaryotes, respectively.

**Figure 3:**
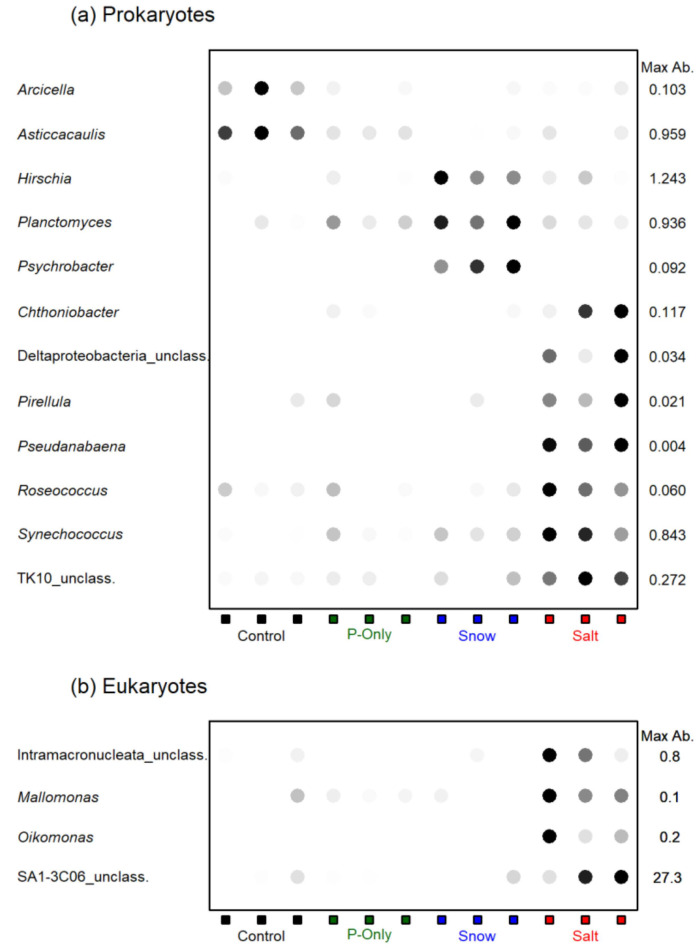
Taxa that increased in response to the different treatments. Max. ab.: highest abundance, expressed as a percentage of the total number of reads. The shading scale for the dots goes from 0 (white) to Max Ab. (black). (**a**) prokaryotes; (**b**) eukaryotes.

The Salt and Snow treatments formed two distinct clusters, indicating that the microbial community responded differently to the chloride supplied alone versus at the same concentration in the snowmelt cocktail (Figure 2). Seven bacterial genera, representing a total of approximately 6% of the community reads, increased in relative abundance in the Salt treatment (ANOVA and Tukey’s HSD, Figure 3). The most abundant was the cyanobacterium *Synechococcus* sp. (0.63%), along with *Chtoniobacter* sp. (0.07%), and *Pirellula* sp. (0.01%). The filamentous cyanobacterium *Pseudanabaena* sp. was only detected in the Salt treatment (ANOVA; *F* = 235.6; *p* < 0.01). Other salt-stimulated genera were found in all the other treatments, but at 2–10-fold lower relative abundance (Table 3). In the Snow treatment, some prokaryotic taxa showed an increase in relative abundance, notably the psychrophilic/psychrotolerant genus *Psychrobacter* (present only in low abundance in the lake; approximately 0.001% of total reads), *Hirschia* sp., and *Planctomyces* sp. These taxa collectively represented <2% of the total community reads. For the eukaryotes, the most pronounced change was that of an unclassified cryptophyte in the SA1-3C06 clade, which represented approximately 1% of the community reads in the other treatments, but become co-dominant in the Salt treatment with a mean relative abundance of 18% (Figure 3, Table 4). *Mallomonas* also responded to chloride addition (Figure 3, Table 4) with a 20-fold increase in abundance, but still represented less than 1% to the total reads. The chrysophyte *Oikomonas* (Table 4) was detected only in the Salt treatment. There were no significant differences among the Control, P-Only and Snow treatments in any eukaryotic taxon.

## 4. Discussion

This study aimed to evaluate the effects of an environmentally relevant concentration of NaCl on a lake microbial community when supplied alone (“Salt”) or in combination with urban snowmelt (“Snow”). In accordance with our hypotheses, the added NaCl induced changes in the microbial community, and the effects differed markedly between the Salt and the Snow treatments. This could be due to chemicals other than NaCl present in the snow, with direct effects on the community or via alteration of the response to NaCl, or to the effects of a microbial inoculum present in the snow. Our results suggest a combination of these effects.

For the prokaryotes, the Snow treatment led to the increased abundance of three taxa, one of which is *Psychrobacter.* This psychrophilic or psychrotolerant genus has been associated with high salinities and anthropogenic disturbance [26] and, because it was not detected at the start of the experiment but was characteristic of the Snow treatment, was likely derived from the snow itself. However, although the road snow might have contributed to the different prokaryotic responses to the Salt and Snow treatments, it was an unlikely source of eukaryotes. There was no change in the eukaryotes associated with the Snow nor the Control, suggesting that their significant response to the Salt treatment had been offset by other chemicals in the snow.

Two factors that decrease chloride toxicity and that might have been present in the snowmelt are increased nutrients [27,28] and increased alkalinity/calcium e.g., [29]. In the present study, the concentration of dissolved organic carbon, total nitrogen and total phosphorus were similar in the Snow and the Salt treatment, but calcium concentrations and alkalinity were higher (Table 2). Although the difference in alkalinity and calcium between the two treatments was modest as compared with the Simmons [29] study, it may have increased the tolerance of the lake microbiome to the moderate salt stress.

Concerning the changes observed in the community taxonomic composition, it is difficult to make direct comparisons with those observed in previous studies. To date, other studies on the effects of chloride on phytoplankton have been conducted during summer (e.g., [4,30]) and have identified sensitive (e.g., diatom) or tolerant (e.g., bloom forming filamentous cyanobacteria) phyla that are seldom present in Lake Saint-Charles during winter. Another consideration is the low concentration used in this study as compared to previously observed step-changes in community composition (120 and 185 mg Cl^−^ L^−1^ for [4,30], respectively). A 2 week exposure to chloride at 50 mg L^−1^ is unlikely to have severe chronic effects on organisms (e.g., [29]). However, interspecific interactions might influence sodium chloride toxicity [31]. For example, exposure of *Daphnia pulex* to chloride induced the production of stress eggs (ephippia), but not when coupled with the threat of predation by rainbow trout, suggesting that this crustacean did not have sufficient energy to deal with both stressors combined [32]. In addition to predation, the exposure to chloride may also impact bacterivory [33], parasitism [34], and competition [35]. For example, *Raphidocelis subcapitata* outcompeted *Chlorella vulgaris* (two green algae) when they were grown together in controlled conditions, but with increasing salinities, the more halotolerant *C. vulgaris* achieved similar or even higher growth rates [35]. These studies suggest that even at sub-lethal concentrations, chloride may affect interactions between species, with consequences for ecological success. The response of the Lake Saint-Charles microbial community to increased chloride is more likely the result of modified interactions due to differences in sub-lethal tolerance, rather than any niche opening due to mortality of salt-sensitive species.

In the present study, the microbial communities were composed of a few dominants and a large number of rare taxa (‘the rare biosphere’), as is typically found in natural assemblages (Table 2 and Table 3, [36]). All of the taxa that responded positively to the Snow and Salt treatments, for both eukaryotes and prokaryotes, were originally in low relative abundance, highlighting the importance of the rare biosphere as a reservoir of diversity and potential ecosystem resilience. Similar effects have been noted in marine-influenced freshwater pools, where community shifts were attributed to the growth of rare taxa rather than the arrival of new species [37]. Compensatory growth by rare taxa can be observed when dominant taxa are negatively affected by perturbation, leaving a niche open for the success of others [36]. However, the response to small perturbations is more likely to be a shift in species proportions than a complete replacement of the community [38], and ongoing function resilience. For example, a mesocosm study of pesticide impacts on freshwater microbial plankton showed that while the community shifted at fine-scale phylogenetic levels, there was a replacement by taxa in the same genera, and an overall maintenance of metabolic capabilities [39].

Although there can be consistency between small-volume results and whole-ecosystem responses [40], microcosm experiments have well-known limitations. For example, the release from grazing pressure, the effects of the container walls, the change in mixing regime and advection, and the removal from any influence by the lower water column and sediments are all factors that may influence the microbial community and its responses to chemical perturbation. In the case of NaCl exposure, assays based on actual snowmelt mixtures as opposed to a single salt are a step closer to understanding potential environmental responses.

To extend this work, it would be useful to take into account the spatiotemporal separation of chemicals that occur during the melting process and the movement of the runoff from the roads to the receiving waters. When urban snow melts, the dissolved chemicals are mobilized in the runoff (reaching freshwater) and a proportion of chemicals that are adsorbed to particles is left behind. There is also evidence for differential elution among the dissolved chemicals, with chloride leaving the snowpack sooner than other constituents [41,42] including calcium [7], which would delay any offsetting effect. In some road environments, snowmelt may be much more toxic than NaCl added alone because of highly bioreactive pollutants, such as 6PPD-quinone that is released from tires and causes acute mortality in coho salmon [43] and certain other fish species [44].

Controlled laboratory experiments are a valuable approach to evaluate the toxicity of contaminants. However, in the case of a more complex exposure to multiple stressors with landscape scaling questions, as with snowmelt, there is a need for whole-lake or whole-catchment monitoring and analysis. This would address the multiple factors that may influence the magnitude and timing of chemical delivery, and the response of the microbial communities. In the same vein, routine inclusion of calcium, sodium, and chloride measurements along with conductivity in monitoring programs would greatly increase the data available for meta-analysis across a full range of salt-contaminated urban lakes. Many of these lakes are currently monitored for nutrients, harmful algal blooms and drinking water quality, and the addition of complementary ionic measurements would help assess the continuing problem of road salt pollution.

## Figures and Tables

**Table 1:** PCR thermal cycling conditions for the sets of primers 515F/806R (prokaryotes) and 1389F/1510R (eukaryotes).

	515F/806R			1389F/1510R		
Steps	Temperature	Time	Cycles	Temperature	Time	Cycles
	(°C)	(s)		(°C)	(s)	
Initial denaturation	None		NA	98	30	1
Denaturation	94	45	36	98	10	30
Annealing	50	60		52	30	
Extension	72	90		72	30	
Final extension	72	60	1	72	270	1

**Table 2:** Chemical composition of Lake Saint-Charles surface water (0–50 cm) in March 2016, and of the melted urban snow and experimental treatments. The values are the means for triplicate samples (SD as the percent mean in parentheses). Alkalinity is in mg CaCO_3_ L^−1^, phosphorus is in µg L^−1^ and all other values are in mg L^−1^.

Variable	Natural Medium				Treatments							
	Lake (t_0_)		Snow		Control ^a^		P-Only ^a^		Snow ^a^		Salt ^a^	
Alkalinity	15.6	(2)	40.9	(13)	14.7	(2)	14.7	(2)	18.0	(1)	14.7	(2)
Calcium	6.4	(1)	14.8	(23)	6.0	(1)	6.0	(1)	7.3	(1)	6.0	(1)
Chloride	20.0	(9)	17.8	(68)	18.9	(9)	18.9	(9)	49.6	(3)	48.9	(3)
Nitrogen ^b^	0.8	(6)	0.4	(46)	0.7	(6)	0.7	(6)	0.7	(6)	0.7	(6)
Phosphorus ^b^	11.4	(22)	84.3	(46)	10.8	(22)	14.8	(16)	16.5	(15)	14.8	(16)
Potassium	0.7	(2)	0.3	(27)	0.7	(2)	0.7	(2)	0.7	(2)	0.7	(2)
Sodium	11.1	(7)	10.6	(63)	10.4	(7)	10.4	(7)	30.4	(2)	30.1	(2)
Sulfate	4.6	(3)	5.3	(72)	4.3	(3)	4.3	(3)	4.9	(3)	4.3	(3)

^a^ Calculated from added chemicals. ^b^ Total (dissolved + particulate).

**Table 3:** The relative abundance (% of total reads) of the twelve most abundant prokaryote taxa (resolved to genus where possible) within each treatment. Each value is the mean of triplicate incubations (SD as the percent mean in parentheses).

Taxon	Control		Taxon	P-Only		Taxon	Snow		Taxon	Salt	
Comamonadaceae_unclass.	22	(52)	*Roseateles* sp.	12	(31)	Comamonadaceae_unclass.	14	(23)	Comamonadaceae_unclass.	18	(10)
*Roseateles* sp.	15	(34)	Comamonadaceae_unclass.	12	(19)	ML635J-21_unclass.	11	(87)	FamilyI_unclass.	8	(57)
*Polynucleobacter* sp.	8	(85)	*Polynucleobacter* sp.	10	(21)	*Polynucleobacter* sp.	10	(30)	*Polynucleobacter* sp.	6	(17)
*Albidiferax* sp.	5	(13)	*Flavobacterium* sp.	10	(87)	*Roseateles* sp.	9	(16)	NS11-12_marine_group_unclass.	5	(17)
*Rhodobacter* sp.	4	(28)	*Albidiferax* sp.	5	(51)	*Albidiferax* sp.	6	(32)	*Roseateles* sp.	5	(46)
*Flavobacterium* sp.	3	(66)	*Caulobacter* sp.	5	(43)	*Flavobacterium* sp.	4	(38)	*Caulobacter* sp.	4	(36)
*Flectobacillus* sp.	3	(171)	ML635J-21_unclass.	4	(155)	NS11-12_marine_group_unclass.	3	(116)	*Albidiferax* sp.	4	(44)
*Caulobacter* sp.	3	(45)	FamilyI_unclass	3	(124)	Proteobacteria_unclass.	3	(132)	Candidatus *Odyssella*	3	(169)
PRD01a011B	3	(159)	Proteobacteria_unclass	3	(122)	Sphingomonadaceae_unclass.	3	(49)	Rhizobiales_unclass.	3	(57)
*Pseudarcicella* sp.	3	(52)	Myxococcales_unclass.	3	(45)	GKS98_freshwater_group	2	(35)	*Sediminibacterium* sp.	3	(39)
*Ferruginibacter* sp.	2	(39)	*Rhodobacter* sp.	3	(76)	*Caulobacter* sp.	2	(28)	ML635J-21_unclass.	3	(118)
OM27_clade	2	(168)	*Pseudarcicella* sp.	2	(52)	Planctomycetaceae_unclass.	2	(31)	Myxococcales_unclass.	2	(81)

**Table 4:** The relative abundance (% of total reads) of the twelve most abundant eukaryote taxa (resolved to genus where possible) within each treatment. Each value is the mean of triplicate incubations (SD as the percent mean in parentheses).

Taxon	Control		Taxon	P-Only		Taxon	Snow		Taxon	Salt	
*Cyclidium* sp.	30	(86)	*Cyclidium*	27	(38)	*Cyclidium* sp.	23	(87)	*Cyclidium* sp.	21	(55)
Conthreep_unclass.	16	(77)	Conthreep_unclass.	26	(113)	Cercozoa_unclass.	13	(82)	SA1-3C06_unclass.	18	(72)
Chrysophyceae_unclass.	9	(18)	Choreotrichia_unclass	10	(116)	Conthreep_unclass.	10	(60)	A31_unclass.	13	(77)
Choreotrichia_unclass.	8	(170)	Haptoria_unclass.	7	(173)	*Rimostrombidium* sp.	10	(173)	Cercozoa_unclass.	10	(73)
Cercozoa_unclass.	6	(92)	Spirotrichea_unclass	7	(132)	Choanoflagellida_unclass.	6	(131)	Chrysophyceae_unclass.	9	(54)
Choanoflagellida_unclass.	6	(173)	Chrysophyceae_unclass	4	(71)	Chrysophyceae_unclass.	4	(97)	Conthreep_unclass.	9	(79)
Glissomonadida_unclass.	5	(173)	Pedinellales_unclass.	3	(127)	Choreotrichia_unclass.	4	(123)	Choreotrichia_unclass	6	(133)
*Leucocryptos* sp.	2	(83)	*Epipyxis* sp.	2	(173)	A31_unclass.	4	(91)	*Paraphysomonas* sp.	3	(169)
A31_unclass.	2	(138)	*Leucocryptos* sp.	1	(158)	Pedinellales_unclass.	2	(73)	*Epipyxis* sp.	3	(42)
SA1-3C06_unclass.	1	(157)	Cercozoa_unclass.	1	(35)	AMT-15-27-30	2	(173)	*Synura* sp.	1	(72)
Pedinellales_unclass.	1	(39)	A31_unclass.	1	(81)	SA1-3C06_unclass.	2	(170)	Haptoria_unclass.	1	(171)
*Chrysamoeba* sp.	1	(119)	*Bolidomonas* sp.	1	(141)	Spirotrichea_unclass.	1	(121)	*Bolidomonas* sp.	1	(60)

## Data Availability

Data are available in GitHub and the National Center for Biotechnology Information repositories at https://github.com/isaza233/Road_salts_snow_experiment and https://www.ncbi.nlm.nih.gov/bioproject/PRJNA681563 (both accessed on 4 April 2022).
